# Cluster randomised trial of a tailored intervention to improve the management of overweight and obesity in primary care in England

**DOI:** 10.1186/s13012-016-0441-3

**Published:** 2016-05-27

**Authors:** Jane Goodfellow, Shona Agarwal, Fawn Harrad, David Shepherd, Tom Morris, Arne Ring, Nicola Walker, Stephen Rogers, Richard Baker

**Affiliations:** 1 Department of Health Sciences, University of Leicester, 22-28 Princess Road West, Leicester, LE1 6TP UK; 2 Faculty of Health and Life Sciences, Coventry University, Richard Crossman Building, Priory Street, Coventry, CV1 5FB UK; 3 Department of Mathematical Statistics and Actuarial Science, University of the Free State, Bloemfontein, 9300 South Africa; 4 Leicester Clinical Trials Unit, Diabetes Research Centre, University of Leicester, Leicester General Hospital, Gwendolen Road, Leicester, LE5 4PW UK; 5 Saffron Group Practice, 509 Saffron Lane, Leicester, Leicester UK; 6 Clinical Lead for Applied Health Research Northamptonshire Healthcare Foundation Trust, Berrywood Hospital, Northampton, NN5 6UD UK

## Abstract

**Background:**

Tailoring is a frequent component of approaches for implementing clinical practice guidelines, although evidence on how to maximise the effectiveness of tailoring is limited. In England, overweight and obesity are common, and national guidelines have been produced by the National Institute for Health and Care Excellence. However, the guidelines are not routinely followed in primary care.

**Methods:**

A tailored implementation intervention was developed following an analysis of the determinants of practice influencing the implementation of the guidelines on obesity and the selection of strategies to address the determinants. General practices in the East Midlands of England were invited to take part in a cluster randomised controlled trial of the intervention. The primary outcome measure was the proportion of overweight or obese patients offered a weight loss intervention. Secondary outcomes were the proportions of patients with (1) a BMI or waist circumference recorded, (2) record of lifestyle assessment, (3) referred to weight loss services, and (4) any change in weight during the study period. We also assessed the mean weight change over the study period. Follow-up was for 9 months after the intervention. A process evaluation was undertaken, involving interviews of samples of participating health professionals.

**Results:**

There were 16 general practices in the control group, and 12 in the intervention group. At follow-up, 15.08 % in the control group and 13.19 % in the intervention group had been offered a weight loss intervention, odds ratio (OR) 1.16, 95 % confidence interval (CI) (0.72, 1.89). BMI/waist circumference measurement 42.71 % control, 39.56 % intervention, OR 1.15 (CI 0.89, 1.48), referral to weight loss services 5.10 % control, 3.67 % intervention, OR 1.45 (CI 0.81, 2.63), weight management in the practice 9.59 % control, 8.73 % intervention, OR 1.09 (CI 0.55, 2.15), lifestyle assessment 23.05 % control, 23.86 % intervention, OR 0.98 (CI 0.76, 1.26), weight loss of at least 1 kg 42.22 % control, 41.65 % intervention, OR 0.98 (CI 0.87, 1.09). Health professionals reported the interventions as increasing their confidence in managing obesity and providing them with practical resources.

**Conclusions:**

The tailored intervention did not improve the implementation of the guidelines on obesity, despite systematic approaches to the identification of the determinants of practice. The methods of tailoring require further development to ensure that interventions target those determinants that most influence implementation.

**Trial registration:**

ISRCTN07457585

**Electronic supplementary material:**

The online version of this article (doi:10.1186/s13012-016-0441-3) contains supplementary material, which is available to authorized users.

## Background

In 2013, 26 % of men and 24 % of women in the UK were obese (body mass index (BMI) 30 kg/m^2^ or above) and 41 % of men and 33 % of women were overweight (BMI between 25 and <30 kg/m^2^) [1, 2]. National guidelines on obesity were published by the National Institute for Health and Care Excellence (NICE) in 2006 [3] and updated in 2014 [4]. They included recommendations for primary care, but care for obesity is often unsatisfactory [5]. Although around 25 % of adults are obese, the percentage recorded as obese in general practice records in 2013–2014 was only 9.4 % [6], and most had no record of receiving a weight loss intervention during a period of up to 6 years [7].

Disseminating guidelines to health professionals often has little effect in changing clinical practice [8]. A review of trials of dissemination of educational printed educational materials concluded that they may have a small effect on professional practice, although the effect on patient outcomes was uncertain [9]. Experimental studies have shown that the addition to guideline dissemination of strategies such as educational meetings, audit and feedback or patient-mediated interventions can lead to positive effects on professional practice and patient outcomes [10]. However, no consistently effective approach has been identified [11]. A potential explanation is that, in different settings, different factors limit what may be achieved. Such factors may be at the level of individual professionals, (e.g. lack of time, knowledge or skills); at the level of the team (e.g. poor leadership, lack of specific team members) or at the level of the organisation (e.g. structures, resources). They may also relate to patients, including their expectations or beliefs. Such factors have been referred to as barriers, obstacles or enablers, but we refer to them collectively as determinants of practice [12]. Many determinants may be identified, especially if several methods for investigating them are used [12]. It follows that if, from amongst the numerous determinants, those few that have most impact on performance can be selected, guideline adherence might be improved if strategies are devised to address them [13]. This approach is referred to as tailoring, and our recent systematic review of 32 randomised trials of tailored interventions concluded that it could be effective, although the effect was variable, and as yet, the best methods of identifying determinants and choosing strategies to address them have not been identified [14]. The studies included in the review used a variety of methods for identifying determinants and choosing strategies, but they did not provide evidence on which methods of tailoring were more effective, and most did not give detailed descriptions of how tailoring was undertaken.

The Tailored Implementation for Chronic Diseases (TICD) project was a 5-year programme involving five European countries to investigate methods to improve the process of tailoring [13]. Component studies have explored methods to identify and classify determinants [12, 15], match interventions to identified determinants [16] and assess the effectiveness of tailored interventions [17]. In this paper, we report a randomised controlled trial from the final stage of the TICD programme. Our research question was in obese or overweight patients in primary care, does a tailored implementation intervention, in comparison with no intervention, increase the proportion of patients who are offered weight management as described in the NICE guidelines? A process evaluation was also undertaken to investigate the integrity of the intervention.

## Methods

### Trial design

**Fig. 1:**
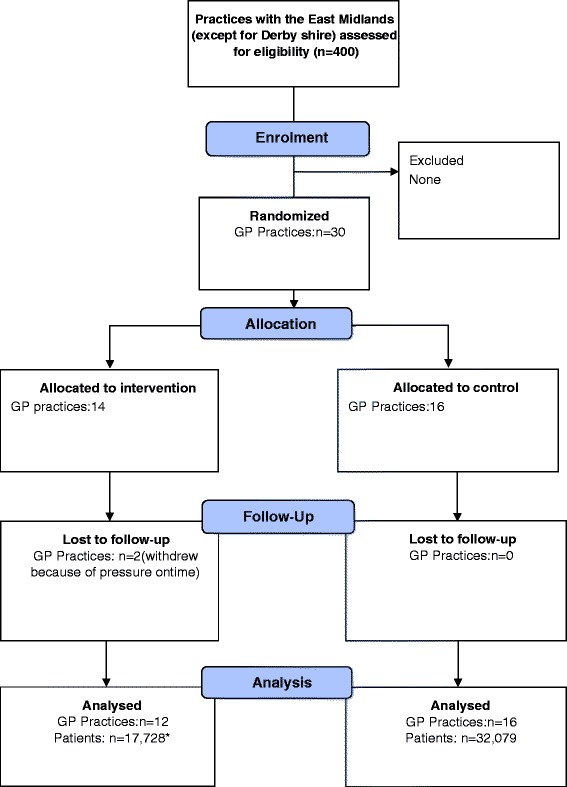
Participant flow diagram

The protocol for the trial has been published [18] (trial registration ISRCTN07457585), and the report of the trial follows the CONSORT statement (Additional file 1) [19]. Research ethics approval was granted from the National Research Ethics Service Committee, Camden & Islington (13/LO/1157). The study is a cluster randomised trial, in which general practices were randomised to two study arms: (1) the study group, in which practices were offered tailored interventions or (2) the control group, in which practices received no intervention (and thus provided usual care). Follow-up was for 9 months. A study participant flow diagram is shown in Fig. 1.

### Participants

With the exception of practices of the Derbyshire Clinical Commissioning Group, all general practices in the East Midlands of England were invited to participate (*n* = 400). Clinical Commissioning Groups are membership organisations comprising groups of practices that share responsibility for commissioning hospital and mental health services. The exclusion criteria for practices were (a) participation in another study of obesity and assessing similar outcomes during the previous year and (b) if the practice had recently changed or were planning to change their computer system over the trial period.

Practice recruitment ceased once the target sample size was reached. Patients were not randomised or directly involved in the study, although their anonymised data were extracted from their electronic medical records. Eligible patients were adults aged 16 years and over in the participating practices who were either overweight or obese at any time during the study period, whether or not they were recorded in the practice obesity register.

### Interventions

The intervention was delivered between November 2013 and January 2014, follow-up in each practice being 9 months after the delivery of the intervention in the practice and completed in all practices by October 2014. The key determinants of practice for each of the four targeted guideline recommendations had been identified in a study involving different practices to those included in the trial, although they were in the same region [12, 18]. The determinants were used to tailor interventions to address each of them (Table 1) [15]. The intervention targeted four key recommendations of the NICE guidelines [3] (Additional file 2) and is described using the TiDieR checklist in Additional file 3 [20]. We did not draw on behavioural theory, relying instead on our own ideas on the strategies most suited to address the determinants, a process informed by the development of the TICD checklist [15]. The checklist included examples of determinants with suggested strategies. For example, professionals were not confident about correctly measuring waist circumference. The TICD checklist advised educational strategies with opportunities to practise necessary skills for determinants related to skills, and we provided this in a session in each practice [15].

**Table 1:** The determinants that were used to tailor interventions

	Recommendation	Determinants	Interventions
1	Determining degree of overweight and overweight	Acceptable ways to raise and discuss the issue with patients (patients’ values in relationship to professional values or those in the recommendation)^a^	Training—presentation, followed by team discussion Model scripts on discussing weight with patients, e.g. ‘Mr X, could we talk about your weight? What are your thoughts about your weight right now?’ ‘Mrs X, I’m concerned about your weight because I think it is causing health problems for you.’
		How to effectively measure waist circumference (The extent to which the targeted healthcare professionals have pre-existing knowledge or expertise about the targeted condition)^a^	Training in waist measurement—information provided, demonstration followed by practise
2	Assessment of lifestyle and willingness to change	Ways to assess willingness to change (patient motivation)^a^	Presentation, discussion, sample questions provided, e.g. ‘What are you goals concerning your weight?’ ‘What changes are you willing to make to your eating or physical activity habits right now?’ ‘What kind of help would you like from me regarding your weight?’ ‘On a scale of 1 to 10, 10 being 100 % ready to take action, how ready are you to lose weight?’
		Resources to motivate and inform (patients’ beliefs or knowledge or ability to learn, or the targeted healthcare professionals’ ability or perceived ability to inform or teach patients necessary knowledge and skills)^a^	Booklet for patients containing clear, proscriptive information (*Weight Loss You Can See*). Posters for practices to invite patients to discuss their weight with a member of the practice team
3	Management of overweight and obesity	Lack of prescriptive information (patients’ beliefs or knowledge or ability to learn, or the targeted healthcare professionals’ ability or perceived ability to inform or teach patients necessary knowledge and skills)^a^	Presentation, provision of summary of the guidelines, and booklet for patients
		Lack of knowledge (the extent to which the targeted healthcare professionals have pre-existing knowledge or expertise about the targeted condition)^a^	Discussion with practices on delegation and the role for practice nurses (an action plan)
4	Referral	Lack of information on referral pathways (the extent to which the resources that are needed to adhere are available)^a^	We collected details on local weight loss and lifestyle services and provided this \]information to practice teams

^a^Definitions of identified determinants given in the checklist [16]

Delivering the interventions involved providing group training to practice teams (GPs, practice nurses and healthcare assistants), including a presentation, discussion and provision of the resources (patient booklets, BMI charts, calories and portions leaflets, posters, information on referral pathways). The training lasted around 1 h.

#### Training and resources for professionals

Training sessions were conducted by a registered dietitian and began with a summary of the guidelines for professionals. Training addressed the issue of sensitively raising and discussing weight with patients, as they may be reluctant to discuss their weight or follow a proposed weight loss intervention [21, 22]. Training in waist measurement was provided with a live demonstration and explanation of the relationship of waist circumference to health risks. In the training session, ways in which the practice managed obese and overweight patients were discussed and the adoption of alternative approaches considered.

Posters for consulting rooms containing information on how to measure waist circumference were given as a visual reminder. Training was given on how to assess patients’ readiness to change their lifestyle and how to calculate energy requirements [23]. Professionals were also provided with example scripts to use in raising and discussing weight with patients. A script containing questions to assess a patient’s motivation and willingness to change were also provided, for use in discussion with patients. They were also given a prescriptive weight loss plan for patients because professionals felt that they did not always have sufficient knowledge or skill to advise patients on changes to their diet.

A poster and associated patient leaflet were provided to help professionals inform patients of the benefits of losing 5–10 % of their weight and to increase patient motivation through showing the benefits of a modest weight loss. Additional posters were also provided in paper and electronic format, including a poster to encourage patients to speak to a professional about their weight, plus BMI charts, and dietary guidance.

At the time of the study, there were various community programmes to improve health and assist weight loss, some of which were available for patients to self-refer into, whilst others required a referral from a professional. Many professionals were not aware of the variety of services available or how to refer patients to them. During the intervention, professionals were asked to list all of the local services they were aware of. After visiting practices, the research team also searched for additional referral options, and we then provided teams with a complete list of local services and referral pathways.

#### Resources for patients

Practices were given materials to help motivate patients, assess lifestyle and patients’ willingness to change and prescriptive information on the management of overweight and obesity. The ‘Weight loss you can see’ information booklet provided patients with visible pictures of portion sizes for everyday foods [23] and a prescriptive energy deficit diet. The energy level prescribed for a patient was based on an estimate of their initial maintenance energy needs minus 600 kcal/day [3], and professionals were given blank diet sheets for patients to record their food intake, and example 1800, 2000 and 2600 calorie diets.

#### Identification of an obesity lead

Each practice team designated a professional to lead on their management of overweight and obesity. We spoke with the local lead prior to delivering the intervention to identify their current management of obesity in order to identify any areas where the intervention needed to be adjusted to meet the needs of the practice. We also encouraged the local lead to take charge in implementing the intervention with their team, and telephoned them regularly (approximately once per month), and undertook at least one further practice visit.

We also worked closely with the obesity lead to improve their knowledge of the care of overweight and obese patients and to identify additional resources and tools which may be useful. We also asked teams during the intervention workshop to discuss barriers within their own practices and ways in which they could be overcome. This led to some local adaptation of the intervention to meet practice needs. For example, one practice suggested that paying for fresh food and gym membership was an issue. In response, we provided a healthy eating on a budget leaflet for use with patients. We also provided additional leaflets for particular groups of patients. These included a leaflet on how diet can affect diabetes, high blood pressure and cholesterol, a leaflet on how food can improve a person’s mood, healthy packed lunches and healthy South Asian food. These leaflets were offered to all the other practices. Another practice was concerned that doctors would be spending more time weighing patients. After discussion, the team decided that the practice nurses and healthcare assistants would attend to most weight management work and refer to the doctor for specific medical advice.

During the monthly telephone calls and additional meeting, we assisted several practices develop links with potentially useful local services, for example, an exercise class for people with limited mobility being run by a volunteer centre, or a health trainer service that offered one-to-one support in weight management. In these telephone calls, we also asked whether practices were having any difficulties, or were using the resources as planned, and when necessary, we addressed concerns in the follow-up visits.

### Randomisation

Randomisation was performed independently by the Leicester Clinical Trials Unit. Randomisation took place at the level of the practice, and the outcomes were measured at the level of the patient, making this a cluster randomised trial. Recruitment ceased once adequate numbers of practices were recruited. Practices were randomised once their expression of interest had been received and were informed afterwards to which group they had been assigned.

Randomisation was stratified by list size (<6000, ≥6000) and deprivation (scores of <20, and ≥20 using the practice Index of Multiple Deprivation [IMD] scores 2010) [24], the cut point for list size being the median list size in England and for IMD, the median of all practices in England. The IMD is a measure used in England to describe socio-economic deprivation, a higher score indicating greater deprivation. It combines indicators in seven domains (income, employment, health, education, housing, crime and environment) into a score for each small area, the practice IMD score being the weighted average of the scores of the areas in which the practice’s patient live. For each of the four strata, a randomisation list (with block size 4) was produced using SAS PROC PLAN with a random seed number. After recruitment of a new practice, the Clinical Trials Unit was provided with list size and deprivation score. The Clinical Trials Unit then communicated the next available treatment code from the randomisation list.

Practices were randomised sequentially. To avoid large imbalances between the four strata (to mimic the underlying distribution of GP practices in England), the following restriction was implemented: none of the four strata were allowed to contain more than nine practices, and the maximum of nine practices was only allowed if all other strata contained at least five practices; otherwise, the maximum should be eight.

#### Blinding

Participant teams could not be blinded to receipt of an intervention. Data collection was blinded and used a standard electronic system that extracted data from the general practice electronic health records and, to minimise bias, all data were collected using full anonymisation using electronic data extraction queries suitable for the different types of general practice computer systems used in England [25].

### Outcomes

#### Primary outcome

The primary outcome was the proportion of overweight or obese patients to whom the health professional had offered a weight loss intervention within the study period. This was operationalised as a record of either advice on weight loss, diet or other diet interventions, advice on physical activity or referral to a weight loss service. This was therefore an inclusive definition of a weight loss intervention, allowing for any record of an attempt to offer help with weight loss. The patient population was defined as all patients with a BMI measurement of 25 kg/m^2^ or higher recorded in their medical notes at any time during the follow-up period or the 9-month baseline period.

#### Secondary outcomes

The secondary outcomes were (1) the proportion of patients with a BMI or waist circumference measurement recorded within the study period; (2) the proportion of patients with a record of lifestyle assessment; (3) the proportion of patients referred to privately or publicly funded external weight loss services, and the proportion managed systematically within the practice, usually by referral to a practice nurse (internal weight management); (4) the proportion of overweight/obese patients who changed weight during the study period and (5) the mean weight change over the same period.

#### Process evaluation

The process evaluation was conducted after implementation of the intervention and completion of the follow-up period. We recruited a purposive sample of professionals from the intervention arm of the trial and undertook semi-structured interviews with an independent researcher not directly involved in the trial. The interviews were conducted by telephone and were recorded after informed consent was given by the respondents and then transcribed. The interviews lasted 30–40 min and examined professionals’ experiences of the intervention. The interview questions are shown in Table 2. We did not directly ask about harms of the intervention, although the interview did permit interviewees to report difficulties or disadvantages. The interviews were analysed using a qualitative content analysis following a mixed deductive-inductive coding approach. A framework for the main categories of the analysis was used that reflected the questions of the process evaluation. The analysis was supported by the use of Atlas.ti [26].

**Table 2:** Questions in the process evaluation interviews

1. What made you participate in the project in the first place? What where your reasons and what were your expectations? 2. Did the implementation program help you to adhere to the recommendations? a) If yes, what components did you find helpful and why? b) If no, why not and what strategies would have been more helpful? 3. Were there any other factors or developments which made it difficult for you or helped you to adhere to the recommendations? 4. Having experienced the program, what would you recommend for the future? You may think of further development, wider implementation or perhaps research.	

### Statistical methods

The primary analysis was conducted at the patient level, using generalised estimating equations, with an exchangeable correlation matrix to account for clustering. The primary outcome was the proportion of patients offered a weight loss intervention, a binary variable, a logit link function being used so that the treatments could be compared by odds ratios. The model was adjusted for the stratification variables (list size, deprivation score). The practices were assigned to treatment groups based on the intention to treat principle. There was no analogue of this at the patient level because no patient protocol deviations were possible.

It was intended to adjust the model in the primary analysis for the outcome at baseline, but the electronic data extraction only collected data for a small part of the baseline period (which varied with each cluster, mean 66 days, rather than the intended 9 months). Therefore, the primary analysis was conducted without adjustment for baseline, but in the light of this limitation, an additional unplanned sensitivity analysis was carried out as follows. A cluster-level summary of the primary outcome was calculated for each cluster (proportion of patients offered a weight loss intervention), and a cluster-level summary of this outcome at baseline was imputed in each cluster using the limited data that had been obtained at baseline. The imputation was done by scaling the proportion up to 9 months (e.g. if the outcome proportion was 4 % collected over 2 months then the imputed outcome at baseline was 4 % × 9/2 = 18 %). The outcome was treated as a continuous variable and was analysed in a cluster-level analysis using a general linear model, adjusted for the stratification variables and the imputed outcome at baseline. Three practices in the control group were excluded from this sensitivity analysis because they had fewer than 30 days of data at baseline, and this time period was judged too short for use in our imputation procedure.

The generalised estimating equation analysis was also conducted with weight at baseline as an additional covariate, as well as with no adjustments. The unplanned cluster-summary analysis was also performed without adjusting for the imputed outcome at baseline.

The binary secondary outcomes were analysed in the same fashion as the primary outcome, including the unplanned sensitivity analyses, as these outcomes suffered from the same data extraction limitation. The continuous secondary outcomes were extracted correctly and these were analysed as planned, in the same manner as the primary outcome except that the link function was the identity link, and the outcome at baseline was included as a covariate.

The follow-up period was defined as the 9month period starting at the date of intervention delivery (intervention group) or the date of randomisation (control group). The baseline period was defined as the same 9-month period in the preceding year.

Descriptive characteristics of the practices and patients at baseline and follow-up were summarised by treatment arm, using mean (standard deviation) or median (interquartile range) for continuous variables as appropriate, and count (percentage) for categorical variables.

### Sample size

We assumed that, in the control arm, the level of adherence to the guideline recommendation on the offer of a weight loss intervention would be 46 %. This estimates was based on a local pilot study of management of obesity in primary care completed in 2010 to 2011 [27] and was measured at the practice level. The aim of the study was to detect an increase to 60 % adherence in the intervention arm with 80 % power, using a two-sided test with alpha of 0.05. The ICC was assumed to be 0.05. We determined the number of clusters per treatment using these values and with various numbers of clusters and cluster sizes (Additional file 4) [28]. Based on these scenarios, a total sample size of 28 practices was selected, which would allow adequate power even in the case of drop out of up to four practices.

## Results

### Recruitment

Thirty practices were recruited, 16 in the control and 14 in the intervention group. Of these, two practices withdrew from the intervention group between randomisation and receiving the intervention because they felt unable to devote the time to the study (see Fig. 1).

### Baseline data

Table 3 shows the practice characteristics at baseline. There were some differences between the intervention and control groups for location, practice size and ethnicity of the patient population. The mean BMI in both treatment arms fell into the obese category, 30.2 kg/m for the control practices and 30.5 kg/m^2^ for the intervention practices.

**Table 3:** Baseline characteristics of participating practices

Practice level	Control (*n* = 16)	Intervention (*n* = 12)
Single handed	1	3
Duo practice	0	0
Group practice	15	9
Rural area	6	3
Urban area	10	9
Deprivation score	24.7 (9.8)	26.2 (12.0)
Practice list size	5968 (3543–13,390)	4065 (2191–7373)
Patient level		
Weight (kg)	[*n* = 20,955] 86.1 (17.9)	[*n* = 12,171] 87.0 (18.1)
BMI (kg/m^2^)	[*n* = 8948] 30.2 (5.4)	[*n* = 4481] 30.5 (5.8)
Waist circumference (cm)	[*n* = 1922] 98.5 (13.0)	[*n* = 818] 101.6 (18.0)
Age	[*n* = 32079] 50.1 (18.6)	[*n* = 17728] 53.4 (17.8)
Sex		
Male	32,538 (47.5 %)	17,675 (47.6 %)
Female	35,969 (52.5 %)	19,476 (52.4 %)
Ethnicity		
White	21,451 (65.6 %)	14,972 (72.9 %)
South Asian	5474 (16.7 %)	2184 (10.6 %)
Black	2055 (6.3 %)	1367 (6.7 %)
Mixed	650 (2.0 %)	399 (1.9 %)
Other	3060 (9.4 %)	1626 (7.9 %)
Comorbidities		
Ischaemic heart disease	2226 (6.9 %)	1405 (7.9 %)
Hypertension	8647 (27.0 %)	5205 (29.3 %)
Disorder of lipid and lipoprotein metabolism	3315 (10.3 %)	1919 (10.8 %)
Cerebrovascular disease	1315 (4.1 %)	857 (4.8 %)
Diabetes	5371 (16.7 %)	3264 (18.4 %)
Highest BMI classification throughout the trial		
Number of patients with a BMI	35,686	19,847
Number overweight or obese	32,079 (89.9 %)	17,728 (89.3 %)
Overweight	17,136 (48.0 %)	8960 (45.1 %)
Obese	14,943 (41.9 %)	8768 (44.2 %)

Numbers presented are *xx* (*xx.x*) = mean (SD) or *xx* (*xx.x*%) = frequency (%) or *xx* (*xx* − *xx*) median (IQR)

### Principal results

#### Primary outcome

Table 4 shows the results of the primary outcome at follow-up. There were no significant differences in the proportion of patients offered a weight management programme between the control and intervention practices (15.1 % in the control practices, 13.2 % in the intervention practices, *p* = 0.53). This result was replicated in the unplanned sensitivity analysis.

**Table 4:** Results of patient-level GEE analyses

	Control	Intervention		Odds ratio	
	(*n* = 32079; 16 practices)	(*n* = 17728; 12 practices)	ICC	OR (95 % CI)	*p* value
Primary outcome					
Weight management	15.1 % (10.8 %)	13.2 % (5.9 %)	0.094	1.17 (0.72, 1.89)	0.53
Secondary outcomes					
BMI or waist circumference measured^a^	42.7 % (10.3 %)	39.6 % (10.6 %)	0.031	1.15 (0.89, 1.48)	0.28
Referral to external weight loss services	5.1 % (3.4 %)	3.7 % (3.4 %)	0.026	1.45 (0.81, 2.63)	0.21
Internal weight management	9.6 % (9.1 %)	8.7 % (6.7 %)	0.123	1.09 (0.55, 2.15)	0.81
Lifestyle assessment	23.1 % (7.6 %)	23.9 % (6.1 %)	0.025	0.98 (0.76, 1.26)	0.88
Weight loss of at least 1 kg^b^	42.2 % (4.1 %)	41.7 % (4.1 %)	0.003	0.98 (0.87, 1.09)	0.67
				Mean difference	
				Mean (95 % CI)	p-value
BMI^c^	30.4 (0.9)	30.5 (1.1)	0.000	0.08 (−0.12, 0.28)	0.43
Weight^b^	85.3 (3.2)	87.5 (1.2)	0.002	0.05 (−0.32, 0.41)	0.81

An odds ratio <1 favours the intervention group. A mean difference >0 favours the intervention group. *xx.x*% (*xx.x*%) = mean (SD)

^a^Control (*n* = 20955), intervention (*n* = 12171)

^b^Control (*n* = 9769), intervention (*n* = 5784)

^c^Control (*n* = 2440), intervention (*n* = 1243)

#### Secondary outcomes

There were no significant differences between the number of patients in the control and intervention practices who had their BMI or waist circumference measured (control 42.7 %, intervention 39.6 %, *p* = 0.28), were referred to external weight loss services (control 5.1 %, intervention 3.7 %, *p* = 0.21), were provided with an internal weight management programme (control 9.6 %, intervention 8.7 %, *p* = 0.81), had a lifestyle assessment (control 23.1 %, intervention 23.9 %, *p* = 0.88) or lost at least 1 kg of body weight (control 42.2 %, intervention 41.7 %, *p* = 0.67). The adjusted means for changes in BMI and weight slightly favoured the intervention group, although there were no significant differences between the control and intervention groups. These results were replicated in the unplanned sensitivity analyses of the binary outcomes.

#### Weight management and lifestyle advice change from baseline

Table 5 shows the change from baseline for weight management and lifestyle advice after adjusting for imputed baseline characteristics. There were no significant changes from baseline for the proportion of patients offered a weight loss intervention (primary outcome). The adjusted mean suggested that there was greater improvement from baseline for the intervention group than the control group which was predominantly as a result of the increase in internal weight management. There were also no significant changes in the proportion of patients with a lifestyle assessment or who were referred to external weight loss services. The proportion of patients with a lifestyle assessment over the course of the study was much larger than at baseline in both the control and intervention practices.

**Table 5:** Cluster-level analysis of weight management variables and lifestyle advice with imputed outcomes at baseline

	Control (*n* = 13)		Intervention (*n* = 12)		Mean difference	
	Baseline (imputed)	Follow-up	Baseline (imputed)	Follow-up	Mean (95 % CI)	*p* value
Weight management	8.8 % (8.5 %)	14.7 % (11.9 %)	5.1 % (2.8 %)	13.6 % (6.4 %)	3.7 (−2.5, 9.9)	0.23
Referral to external weight loss services	4.5 % (5.8 %)	4.3 % (3.0 %)	2.8 % (3.0 %)	3.8 % (3.6 %)	0.5 (−2.2, 3.1)	0.73
Internal weight management	4.2 % (4.0 %)	9.9 % (10.1 %)	2.3 % (2.0 %)	9.0 % (7.1 %)	3.3 (−1.7, 8.3)	0.18
Lifestyle assessment	0.0 % (0.0 %)	23.4 % (8.2 %)	0.9 % (2.1 %)	24.3 % (5.8 %)	−0.2 (−6.9, 6.5)	0.95

The outcomes here are continuous cluster-summary variables (percentage of patients with the outcome) as opposed to patient-level binary variables. *xx.x*% (*xx.x*%) = mean (SD). A mean difference >0 favours the intervention group

#### Predictive variables for adherence to the NICE guidelines

Some predictive variables had a significant impact on the primary and secondary outcomes and were included in statistical models where appropriate. These were selected via a model selection procedure. The results of the predictive variables for the outcomes of this study are shown in Table 6. The primary outcome was the proportion of patients to whom professionals had offered a weight loss intervention. An increase in BMI at baseline of 1 kg/m^2^ led to a 3.4 % increase in the odds of being offered a weight management intervention. Similarly, an increase in age of 1 year led to a 1.7 % increase in the odds of being offered a weight management intervention.

**Table 6:** Predictive variables

Outcome variable	Predictor	Odds ratio (95 % CI)
Weight management	BMI at baseline^a^	1.03 (1.02, 1.05)
	Age^a^	1.02 (1.01, 1.03)
BMI or waist circumference measured	Sex (male)^b^	0.70 (0.57, 0.87)
	Waist circumference [cm]^a^	1.01 (1.01, 1.02)
Referral to external weight loss services	Weight at baseline [kg]^a^	1.01 (1.01, 1.02)
Internal weight management	Sex (male)^b^	1.09 (0.99, 1.20)
	Ethnicity (mixed)^c^	0.71 (0.51, 0.99)
	Age^a^	1.01 (1.00, 1.02)
Lifestyle assessment	BMI at baseline^a^	1.02 (1.01, 1.03)
Weight loss of at least 1 kg	Ethnicity (mixed)^c^	0.45 (0.20, 1.00)
	Weight at baseline^a^	1.02 (1.01, 1.02)
	BMI at baseline^a^	1.03 (1.01, 1.06)
	Age^a^	1.02 (1.01, 1.02)
		Increase in outcome (95 % CI)
BMI	Ethnicity (South Asian)^c^	−0.47 (−0.97, 0.02)
	BMI at baseline^d^	0.74 (0.66, 0.82)
	Age^d^	−0.01 (−0.02, −0.00)
Weight	BMI^d^	−0.17 (−0.22, −0.12)
	Weight at baseline [kg]^d^	0.95 (0.92, 0.97)
	Age^d^	−0.07 (−0.08, −0.06)

^a^Comparison between a binary outcome and continuous predictor. For the proportion of patients offered a weight loss intervention (outcome) and BMI (predictor). The odds ratio of 1.03 implies that an increase in BMI by a unit of 1 leads to a 3 % increase in the odds of receiving a weight management intervention

^b^Comparison between a binary outcome and categorical predictor. For example, the odds of having a BMI or waist circumference measured is 30 % lower in men compared to women

^c^Comparison between a continuous outcome and categorical predictor. For example, being of a South Asian ethnicity leads to an increase in BMI of −0.47 (i.e. a decrease of 0.47) in a South Asian when compared to a White European

^d^Comparison between a continuous outcome and continuous predictor. For example, an increase in BMI at baseline of 1 leads to an increase in BMI at follow-up of 0.74

Predictive variables also had a significant impact on the secondary outcomes. Patients were 29.7 % more likely to have a BMI or waist circumference measured if they were female instead of male. The odds of patients being referred or offered an internal weight management programme was 9.3 % higher for men in comparison to women and 28.9 % lower for patients of mixed ethnicity in comparison to white European patients. An increase in BMI of 1 was associated with a 2.3 % increase in patients being provided with a lifestyle assessment. Being of South Asian ethnicity was associated with a decrease in BMI of 0.474 compared to White Europeans. An increase in BMI at baseline of 1 was associated with an increase in BMI at follow-up of 0.740.

#### Process evaluation

The intervention sessions in practices were attended by a total of 78 professionals (mean 6.5/practice, range 2–12). In the process evaluation, 11 professionals (1 GP, 7 practice nurses and 3 health care assistants) were interviewed. Two findings emerged from the interviews, an increase in confidence in managing obesity and appreciation of the resources provided to teams.

Respondents reported feeling more confident about managing obesity. They felt the training increased their weight management knowledge and skills, and felt more confident discussing weight with patients, and better able to manage obese/overweight patients:

I genuinely think the intervention was well received by the practice. (GP).

I just think it’s been helpful for me to be able to speak to patients about; it’s really quite difficult for the likes of me to say to somebody that you are obese, that you are big, so something visually I can draw their attention to without actually having to say you’re fat and you got to do something about it and bring it down. (health care assistant)

The respondents also felt the intervention provided practical resources for use with patients:

I think it is something that actually conceptualises weight loss in a very patient friendly realistic, real life type of way which is digestible by people. (GP)

Majority of the patients really appreciated the literature, they appreciated that it wasn’t just a leaflet given to them, there was follow-up. (practice nurse)

Respondents reported that the *Weight loss you can see* patient booklets [24] provided clear, pictorial guidance on portion sizes and gave patients a clear understanding of appropriate portion sizes. Some respondents felt the printed resources should have been available in different languages and cover a greater variety of ethnic foods:

To be translated but you’d also need to look at the foods for that particular culture. (practice nurse).

The interviews did not disclose harms of the intervention. Some practices reviewed their systems for managing obesity. This nurse set up a group session to use time efficiently and found distributing the information to several patients at once worked well:

I wanted an idea of having a group of say up to ten patients go through all of this with them in a spiel, you know do like a thirty minute, twenty minute presentation and then make sure I got time to bring them in or get somebody to weigh them, height them and work out you know a calorie thing for them to continue. (practice nurse).

## Discussion

### Summary of findings

Our findings are essentially negative. The data presented in Table 4 indicate that there were no improvements in guideline adherence in the intervention group in comparison with the control group. This finding applies to all study outcomes. Our process evaluation, in contrast, suggested that professionals felt more confident in their ability to manage obesity and they found the resources practical.

### Strengths and limitations

We were successful in recruiting an adequate number of practices, but included only a small proportion of all those in the region, and therefore, the practices in the study are likely to unrepresentative of the level of interest of most practices in the management of obesity.

For the primary analysis, our assumption that 46 % of overweight/obese patients would have been offered a weight management intervention at baseline was a huge overestimate, the proportion observed in the study being only 7 %. This would have allowed us to detect a smaller difference in percentage than the 14 % specified in the protocol. In addition, the ICC for the primary outcome was larger than anticipated, at 0.08 as opposed to the assumed 0.05. This would have adversely affected the power, but the ICCs for many of the secondary outcomes were lower than anticipated and these outcomes also failed to display significant differences. There are no reasons to suspect contamination between study groups; the participating practices did not know which practices were in the other study group, excluding three practices that were part of a commercial provider of primary care services. Furthermore, the practices were widely dispersed, and opportunities for the practices to interact, other than via the researchers delivering the intervention, were minimal.

A limitation was the failure of adequate data extraction for the binary outcomes at baseline. However, based on the sensitivity analyses that utilised the baseline data that were extracted, it seems unlikely that these data would have had an impact on the results had a full 9 months of data been extracted, rather than the mean of 2 months. We collected data from electronic records, and it is possible that some actions by professionals were not recorded. The computer systems lacked a standard coding template for obesity management, and we provided practices with a list of codes for documenting the care of overweight and obesity, but despite this, it is likely that some care was not recorded. However, we cannot identify a reason why the extent of recording failures should differ according to study group and therefore do not believe that this is an explanation for our findings.

During the course of the study, incidental initiatives to improve obesity care cannot be ruled out. Although both intervention and control practices were exposed to such initiatives, our intervention was unable to encourage adherence over and above the general pressure to address obesity emanating from health service policy. An additional factor may have been the publication of an update of the NICE obesity guidelines during the course of the study. However, the new guideline did not make substantive changes to the recommendations for primary health care teams.

The duration of the study may have been a limitation. The 9-month period allowed for follow-up would have reduced the numbers of people with obesity who could have visited their practice twice for any weight change to be documented, for example. Also, the response of professionals to the intervention may require several months before they routinely follow the guideline recommendations.

We did not undertake a pilot test of the intervention, but it is possible a pilot test would have revealed weaknesses in our intervention. Pilot testing may be advisable in future studies of tailored interventions. We did not base our intervention strategies on specific behavioural theories, and it may be thought that a theory-driven intervention would have been more effective. However, our review of trials of tailored interventions failed to demonstrate any advantage to use of theories [14]. Empirical evidence that demonstrates the advantages of explicit use of theory is required.

### Interpretation

Since limitation of study design and conduct appear unlikely to explain the negative outcome, other explanations need to be considered. Obesity and overweight may be particularly challenging for primary care teams [29, 30]. The problem is extremely common and is not always perceived by patients as a priority. Easy steps that patients can take are limited—it can be difficult to change personal lifestyle and dietary habits. Services to support overweight and obese people are limited, and weight reduction and exercise services often require a financial contribution from patients. Thus, patient motivation and barriers to access present professionals with additional difficulties that need to be overcome.

General practice is under great pressure consequent upon the ageing population and growing levels of multi-morbidity [31]. Primary care teams may find themselves having to prioritise their activities and may be too busy caring for those who, for example, already have type 2 diabetes to be able to devote much time to people who are overweight or obese. Our intervention might have been strengthened if we had offered support in making additional staff time available for managing obesity.

A further potential explanation for our finding is that our intervention did not identify the important determinants of practice from amongst the many detected [12]. For example, the educational components of the intervention may have had little effect. A review of trials of educational interventions concluded that their effect is likely to be small [32]. Other studies have shown that tailored implementation can lead to improved performance. For example, tailored patient education materials plus decision support and reminders for GPs improved the use of antibiotics for sore throat [33], and tailored educational outreach plus audit and feedback led to improvements in prescribing of antihypertensive drugs [34].

Our intervention involved training with active discussion, provision of scripts, and various resources, delivered in one session to practice teams, although with some additional follow-up. It is possible that the development of skills to discuss obesity and deliver effective management amongst professionals requires a more intensive package of activities to bring about change in performance. Our analysis of the determinants of practice did not suggest that reminders were required, but perhaps, they might have encouraged professionals to begin to discuss the management of obesity with patients more often. However, a more intensive intervention would consume more time and other resources and might therefore not be feasible in many health systems.

### Implications for tailored implementation

Our findings should not be generalised to all tailored implementation methods [15], but they indicate that we have some way to go before we can draw on reliably effective approaches. Researchers should be encouraged to use systematic approaches to identifying determinants and designing interventions and should report the rationale for the chosen interventions. A particular problem is how the most important determinants should be identified from amongst the many uncovered. In addition, ways to extend the variety of interventions that can be used should be explored; for example, interventions that address time pressures, financial disincentives and administrative constraints should be explored, perhaps in association with health service managers and policymakers.

## Conclusions

Despite undertaking a detailed investigation of the determinants of practice, our tailored intervention failed to improve the implementation of the guideline on obesity. Tailored implementation methods require further development before they can be relied upon to be routinely effective.

## Additional files

Additional file 1: CONSORT checklist. (DOCX 24 kb)

Additional file 2: The NICE guideline recommendations for adults for primary care teams. (DOC 23 kb)

Additional file 3: Description of the intervention using TIDieR checklist [18]. (DOC 36 kb)

Additional file 4: Study power depending on number of clusters and average cluster size (UK). (DOC 28 kb)
