# Supplementary material for: Cluster randomised trial of a tailored intervention to improve the management of overweight and obesity in primary care in England
Source: Implement Sci. 2016 May 27;11:77. doi: 10.1186/s13012-016-0441-3 (PMC4884420; doi:10.1186/s13012-016-0441-3)
Supplement: Supplementary file 2 — The NICE guideline recommendations for adults for primary care teams. (DOC 23 kb) [file 13012_2016_441_MOESM2_ESM.doc]

**Appendix 2: The NICE guideline recommendations for adults for primary care teams**

1. Determining the degree of overweight or obesity: Healthcare professionals use BMI and/or waist circumference to classify the degree of overweight or obesity. Patients should be told their classification, and how this affects their risk of long-term health problems.
2. Assessment of lifestyle, co-morbidities and willingness to change: A patient’s risk factors, co-morbidities, any presenting symptoms and underlying causes of overweight and obesity need to be assessed. Their motivation and willingness to change is extremely important.
3. Management of overweight and obesity: A multi-component intervention should be offered to encourage increased physical activity, improved eating behavior, and healthy eating. Drugs may be used in certain groups. The intervention should involve long-term follow up by a trained professional and be tailored to the patient’s preferences, initial fitness and lifestyle.
4. Referral: This is appropriate when the cause is uncertain, if conventional treatment has failed and surgery is being considered or there are complex co-morbidities and specialist intervention are needed.
