# Supplementary material for: Cluster randomised trial of a tailored intervention to improve the management of overweight and obesity in primary care in England
Source: Implement Sci. 2016 May 27;11:77. doi: 10.1186/s13012-016-0441-3 (PMC4884420; doi:10.1186/s13012-016-0441-3)
Supplement: Supplementary file 3 — Description of the intervention using TIDieR checklist [18]. (DOC 36 kb) [file 13012_2016_441_MOESM3_ESM.doc]

**Appendix 3. Description of the intervention using TIDieR checklist [18]**

|  |  |
| --- | --- |
| 1. Name of intervention | Tailored implementation |
| 1. Rationale | The performance of health professionals is believed to be influenced by various factors, which may either impede or enhance performance. For example, lack of skills would impair performance. These factors are collectively referred to as determinants, and the rationale or theory of tailored implementation is that, if an implementation intervention is tailored to address the determinants of practice, performance will improve. |
| 1. Materials | The investigation of determinants involved interviews of health professionals and patients. The tailoring – the selection of intervention components to address the identified determinants – was undertaken by groups of professionals and implementation experts.  The tailored intervention involved a one hour workshop to the practice team (GPs, practice nurses, health care assistants). It involved presentation of the guideline recommendations, discussion of the practice’s current approach and ways of improving weight management, development of skills (waist circumference measurement, talking with patients on obesity), provision of didactic information for patients on diets, and information on local weight and fitness services. After the workshop, there were monthly telephone calls and at least one further visit to each practice. |
| 1. Procedures | Investigation of determinants – interviews of general practitioners, practice nurses and patients. The following determinants were identified: acceptable ways to raise and discuss the issue with patients; How to effectively measure waist circumference; ways to assess willingness to change; resources to motivate and inform; lack of prescriptive information; lack of knowledge; lack of information on referral pathways.  Tailoring- the research team, informed by a checklist of determinants and suggested strategies[16] identified strategies to address each determinants  Intervention delivery – the strategies were delivered in the workshop, and in subsequent monthly contact with each practice team. Resources for professionals and patients were provided. |
| 1. Providers | Investigation of determinants – we interviewed health professionals and patients, and conducted a survey.[13]  Tailoring – we organised group sessions of health professionals and implementation experts to discuss the determinants and suggest strategies.[17]  Intervention delivery – this was led by a research dietician, supported by an additional researcher. |
| 1. Modes of delivery | Investigation of determinants – interviews, focus groups, etc  Tailoring – group sessions.[17]  Intervention delivery – face to face meetings at practices |
| 1. Where | Investigation of determinants- undertaken in general practices in the East Midlands of England  Tailoring - in an academic centre in the East Midlands of England.  Intervention delivery was to general practices. We contacted practices at least once per month after the delivery of the intervention workshop, and conducted at least one follow up visit to each practice. |
| 1. When and how much | Intervention delivery – the initial workshop lasted around one hour |
| 1. Adaptation | Did we adapt for different practices? Some practices requested additional resources, for example, leaflets for particular patient subgroups. We provided these, and also offered the additional resources to the other intervention group practices. |
| 1. Modifications | The intervention was not modified during the course of the study |
| 1. Fidelity | During the monthly telephone calls and follow up visit, we discussed with practices their activities in managing obesity and their use of the resources provided. We clarified which resources they were using, and ask them to demonstrate how they were using them to check they were using the resources correctly. |
| 1. Extent to which delivered as planned | Identification of determinants – we did identify determinants, but we cannot be sure if they were the key ones  Tailoring – the approach was not piloted or tested; it was not based on an underlying theory, but based on opinion and experience of health professionals and implementation experts; we cannot be sure the determinants were addressed by interventions most likely to improve performance.  Delivery - Interviewees comments in the process evaluation indicated regular use of the resources provided, and greater confidence in their skills in managing obesity. |
