# Supplementary material for: Cluster randomised trial of a tailored intervention to improve the management of overweight and obesity in primary care in England
Source: Implement Sci. 2016 May 27;11:77. doi: 10.1186/s13012-016-0441-3 (PMC4884420; doi:10.1186/s13012-016-0441-3)
Supplement: Supplementary file 4 — Study power depending on number of clusters and average cluster size (UK). (DOC 28 kb) [file 13012_2016_441_MOESM4_ESM.doc]

**Appendix 4:** Study power depending on number of clusters and average cluster size (UK)

| Average cluster size | >1,130 | 163-212 | 213-309 | 310-568 | 569-3,464 | >3,464 | >287 |
| --- | --- | --- | --- | --- | --- | --- | --- |
| Power (1-ß) | 0.80 | 0.80 | 0.81 | 0.82 | 0.83 | 0.84 | 0.85 |
| Number of clusters | 12 | 13 | 13 | 13 | 13 | 13 | 14 |
